# Bridging treatment prior to liver transplantation for hepatocellular carcinoma: radioembolization or transarterial chemoembolization?

**DOI:** 10.1186/s40001-022-00708-w

**Published:** 2022-05-26

**Authors:** Tamás Benkö, Julia König, Jens M. Theysohn, Clemens Schotten, Fuat H. Saner, Jürgen Treckmann, Sonia Radunz

**Affiliations:** 1 Department of General, Visceral and Transplant Surgery, University Hospital Essen, Essen, Germany; 2 Department of Diagnostic and Interventional Radiology and Neuroradiology, University Hospital Essen, Essen, Germany; 3 Department of Gastroenterology and Hepatology, University Hospital Essen, Essen, Germany

**Keywords:** Bridging treatment, Hepatocellular carcinoma, Liver transplantation, Radioembolization, Transarterial chemoembolization

## Abstract

**Background:**

In hepatocellular carcinoma (HCC) patients, intraarterial therapies are regularly employed as a bridge to liver transplantation to prevent tumor progression during waiting time. Objective of this study was to compare HCC recurrence after liver transplantation following TACE or radioembolization bridging treatment.

**Methods:**

We retrospectively analyzed prospectively collected data on 131 consecutive HCC patients who underwent liver transplantation between January 2007 and December 2017 at our liver transplant center (radioembolization *n* = 44, TACE *n* = 87). Multivariable logistic regression and cox proportional hazard regression models were used to evaluate factors associated with tumor recurrence and post-transplant survival.

**Results:**

Between groups, patients were comparable with regards to age and gender. In the radioembolization group, Milan criteria for HCC were met significantly less frequently (20.5% vs. 65.5%, *p* < 0.0001). Patients in the radioembolization group required significantly fewer intraarterial treatments (1 [1–2] vs. 1 [1–7], *p* = 0.0007). On explant specimen, tumor differentiation, microvascular invasion and tumor necrosis were comparable between the groups. HCC recurrence and overall survival were similar between the groups. Multivariable analysis detected increasing recipient age, male gender, complete tumor necrosis and absence of microvascular invasion being independently associated with decreased odds for HCC recurrence. Increasing model of end-stage liver disease (MELD) score and tumor recurrence were independently associated with increased odds of post-transplant death.

**Conclusions:**

Intraarterial bridging treatment leading to tumor necrosis may not only prevent waitlist drop-out but also facilitate long-term successful liver transplantation in HCC patients. Both radioembolization and TACE represent potent treatment strategies.

## Background

Worldwide, hepatocellular carcinoma (HCC) is the sixth most common malignancy and the third most frequent cause of cancer-related mortality [1]. HCC develops predominantly in patients with underlying chronic liver disease. Surgical treatment of HCC lesions is only possible in well-compensated patients without severe portal hypertension due to the high incidence of perioperative morbidity and mortality in patients with advanced cirrhosis.

For HCC patients with end-stage liver disease, liver transplantation has become the treatment of choice. Liver transplantation affords an opportunity both to cure HCC and to prevent future de novo HCC through the removal of the cirrhotic liver, the main risk factor for hepatocarcinogenesis [2]. Nowadays, HCC patients have the highest rates of waitlisting for liver transplantation among all candidates with end-stage liver disease [3]. Yet, shortage of donor organs has resulted in an overall increase of waiting time with an increased risk of waitlist dropout due to tumor progression in HCC patients. According to an international consensus conference held in 2010, locoregional bridging treatment should be considered in patients expected to wait more than 6 months to diminish waitlist dropout because of tumor progression [4]. Since the actual waiting time to liver transplantation is difficult to predict, the application of locoregional bridging treatments has become standard of care in many liver transplant centers. Treatment should be selected according to Barcelona Clinic Liver Cancer (BCLC) standard [5].

The two main intraarterial techniques used in the treatment of HCC are transarterial chemoembolization (TACE) and radioembolization. So far, no recommendation can be made for preferring any particular type of intraarterial locoregional treatment prior to liver transplantation [4, 6, 7]. TACE as the most frequently applied intraarterial bridging treatment in liver transplant candidates is currently delivered directly by means of super-selective catheterization into the hepatic arterial branches feeding the tumors [5, 6]. Treatment repetitions at regular intervals or ‘on-demand’ are required; however, repeated TACE may cause ischemic injury to non-tumoral liver tissue.

In general, radioembolization using the radionuclide yttrium-90 is employed in the control of HCC when TACE cannot be applied because of multifocality and/or large size of lesions or in the setting of portal vein thrombosis [8–10]. Radioembolization has been shown to be better tolerated and associated with fewer treatment sessions [11]. Despite radioembolization being traditionally reserved as a salvage treatment for locally advanced HCC, both large cohort retrospective as well as prospective randomized studies suggest that radioembolization may potentially be superior to TACE as bridging treatment to liver transplantation. Radioembolization demonstrates higher complete response rates and longer time-to-progression compared with segmental chemoembolization for HCC, with similar or better toxicity profiles [12, 13]. Thus, many international centers have begun to embrace radioembolization as a first-line treatment for hepatocellular carcinoma [14, 15].

In addition to prevent tumor progression during waiting time, response to bridging treatment may provide insight into the biological behavior of tumors and serve as another selection criterion for liver transplantation candidacy [16]. Sustained response to locoregional bridging treatment over a period of time has been proposed as a surrogate marker of more favorable tumor biology [17–19]. The risk of HCC recurrence after liver transplantation is significantly higher in patients without response to locoregional treatment, regardless of Milan status prior to liver transplantation [20, 21]. Especially patients achieving complete pathologic response have been shown to have significantly superior recurrence-free survival [7].

The real impact of different types of locoregional treatment prior to liver transplantation is still in debate. Gabr et al*.* presented United Network for Organ Sharing (UNOS) data on long-term outcomes of transplanted patients who had undergone bridging treatment consisting of TACE or radioembolization [22]. So far, no European data are available. Owing to differences in waiting time, priority allocation and donor organ quality there is considerable interest in the analysis of such European data. Thus, primary objective of this study was to analyze the rate of tumor recurrence among patients who underwent either radioembolization bridging treatment or TACE bridging treatment. The comparison of these two therapies could help to better define the treatment strategy in intermediate/advanced HCC patients awaiting liver transplantation.

## Patients and methods

Data on 131 patients who underwent liver transplantation for HCC after bridging treatment consisting of either radioembolization or TACE at our liver transplant center between January 2007 and December 2017 were retrospectively analyzed. The study was approved by the local ethics committee and was conducted in accordance with the Helsinki Declaration of 1975, as revised in 2008. Inclusion criteria were all adult, first-time liver transplant recipients. Patients who had to be removed from the transplant waitlist because of progressive disease despite bridging treatment had been excluded from the study (*n* = 6 after radioembolization, *n* = 8 after TACE). Primary outcome was treatment response as measured by pathologic tumor response on explant specimen. Secondary outcomes were tumor recurrence and survival.

All patients were assessed at a multidisciplinary transplant and liver tumor conference. A unanimous consensus was reached that each patient would undergo intraarterial bridging treatment to avoid tumor progression during waiting time. Patients with a single lesion ≤ 7 cm or up to three lesions ≤ 5 cm were scheduled for TACE, while patients with lesions > 7 cm, more than three lesions or multiple tumor feeding arteries were scheduled for radioembolization. The Milan criteria were assessed prior to bridging treatment as only pre-treatment tumor criteria are considered for organ allocation in Germany.

Selective or super-selective TACE procedure was performed injecting the chemotherapy emulsion (Lipiodol, Mitomycin C) into the distal tumor-feeding arteries followed by temporary embolization with gel foam to obtain complete stasis in the tumor-feeding vessels. TACE treatment was repeated ‘on-demand’ for patients with residual or recurrent tumors [23]. Radioembolization was delivered via glass microspheres containing the radionuclide Yttrium-90 administered at the lobar or segmental level within the right or the left hepatic artery. Sequential bilobar treatment was performed within 4 weeks if indicated [24]. Every type of intraarterial treatment was with the intent to bridge rather than to achieve downstaging. In Germany, patients who had once been outside the Milan criteria are not eligible to receive a MELD upgrade even if downstaging is achieved. Pre-treatment AFP was categorized as ≤ 20, 21–100, and > 100 IU/mL [25, 26].

Patients underwent orthotopic liver transplantation without veno-venous bypass when a suitable organ offer became available. Immunosuppression consisted of tacrolimus 0.1 mg/kg adjusted to a trough level of 5–7 ng/mL, mycophenolate mofetil 1 g bid and prednisolone 20 mg, tapered and withdrawn within 6 weeks. Tumor response of the treated lesions on explant pathology was classified as follows: complete necrosis (100% necrosis, absence of any viable tumor cells), partial necrosis (> 50% necrosis, clusters of viable tissue), viable tumor cells (< 50% necrosis) [27]. Routine follow-up involved CT scans as well as AFP tests at month 6, 12 and 24. Thereafter, yearly ultrasounds as well as alpha-fetoprotein (AFP) tests were performed.

All data were tested for normality using the D'Agostino&Pearson omnibus normality test. Categorical variables are presented as percentages and continuous variables as median [range], unless stated otherwise. Differences were tested using chi-square test or Mann–Whitney tests, as appropriate. Patient survival and rate of tumor recurrence were evaluated using the Kaplan–Meier method and compared with the log-rank test. The reference point for all calculations of survival was the day of liver transplantation. A *p* value ≤ 0.05 (two-tailed) was considered to be significant.

Variables clinically relevant to the development of recurrent HCC were included in a binary logistic regression model to estimate the impact of selected variables on tumor recurrence. All analyses have to be regarded as exploratory as we did not adjust the significance level globally in terms of the multiple testing problem. Final variables incorporated in the logistic regression model included recipient age, gender, AFP, Milan criteria, TACE treatment, tumor grading, microvascular invasion and tumor necrosis on explant specimen. Cox regression analysis was performed to assess the relationship between survival time and covariates. Final variables incorporated in the model included recipient age, gender, MELD score, DRI, TACE treatment and tumor recurrence.

Data collection and statistical analysis were performed using Microsoft Excel 2010 (Microsoft Corporation, Redmond, WA, USA) and GraphPad Prism version 6.07 for Windows (GraphPad Software, San Diego, CA, USA). Logistic regression analysis was performed using IBM SPSS Statistics (version 23.0 for Windows, SPSS, Inc., Chicago, IL, USA).

## Results

### Study population

The study population comprises 44 patients receiving radioembolization bridging treatment and 87 patients receiving TACE bridging treatment. The recipient and donor characteristics of both groups are shown in Table 1. There were no differences with regards to recipient age, gender and pre-treatment AFP. In the radioembolization group, hepatitis C associated liver cirrhosis was significantly less common (25.0% vs. 50.6%, *p* = 0.0053). Significantly fewer HCC lesions met the Milan criteria in the radioembolization group (20.5% vs. 65.5%, *p* < 0.0001). Donor characteristics as represented by DRI were comparable between the groups.

**Table 1:** Patients’ and donor characteristics

	Radioembolization (*n* = 44)	TACE (*n* = 87)	*p*
Age (years)	60 [37–70]	59 [29–69]	0.1558
Male gender (%)	84.1	72.4	0.1915
MELD score	12 [6–40]	10 [6-30]	0.0204
Etiology of liver disease			0.0434
Alcohol (%)	27.3	23.0	
Hepatitis B (%)	18.2	13.8	
Hepatitis C (%)	25.0	50.6	
NASH (%)	18.2	6.9	
Other (%)	11.3	5.7	
Within Milan criteria (%)	20.5	65.5	< 0.0001
AFP pre-treatment (IU/mL)	22.5 [2.1–13,923.0]	14.9 [1.5–9860.0]	0.5369
Number of treatments	1 [1, 2]	1﻿ [1-7]	0.0007
Donor risk index	1.740 [1.082–2.484]	1.680 [0.9420–2.992]	0.2734

### Explant specimen

Complete necrosis was detected in 59 (45.0%) explanted liver specimen, partial necrosis in 50 (38.2%) specimen and in 22 (16.8%) specimen no significant necrosis was detected at histopathological examination. The rate of complete necrosis was comparable between both groups (radioembolization group 40.9%, TACE group 47.1%, *p* = 0.5783). Poor differentiation of the tumor, i.e., tumor grading G3, was evident in 14 (10.7%) specimen (radioembolization group 15.7%, TACE group 9.2%, *p* = 0.5506). Microvascular invasion was present in 14 (10.7%) specimen (radioembolization group 18.2%, TACE group 6.9%, *p* = 0.0705). In 6 specimen, both poor differentiation and microvascular invasion was detected. Radioembolization and TACE had each been applied in 3 of these patients (radioembolization group 6.8%, TACE group 3.5%, *p* = 0.4029).

### Tumor recurrence

Twenty (15.3%) patients were diagnosed with recurrent HCC. Time from liver transplantation to tumor recurrence was 14 [4–56] months. Rate of tumor recurrence was higher in the radioembolization group, but this did not reach statistical significance (radioembolization group 23% (*n* = 10), TACE group 11% (*n* = 10), *p* = 0.1224).

When comparing patients with and without tumor recurrence, there were no statistical differences regarding gender, etiology of liver cirrhosis, MELD score, and Milan criteria (Table 2). Patients with recurrent HCC were of significantly younger median age (55 vs. 59 yrs., *p* = 0.0255) and had significantly higher median pre-treatment AFP levels (101.3 vs. 14.2 IU/mL, *p* = 0.0036). On explant specimen, poor differentiation (25.0% vs. 8.1%, *p* = 0.0402) and microvascular invasion were significantly more frequent (45.0% vs. 4.5%, *p* < 0.0001), while complete tumor necrosis was significantly less frequent (5.0% vs. 52.3%, *p* < 0.0001).

**Table 2:** Patients’ and tumor characteristics among recipients with and without recurring hepatocellular carcinoma

	Tumor recurrence (*n* = 20)	Recurrence-free (*n* = 111)	*p*
Age (years)	55 [29–64]	59 [34–70]	0.0255
Male gender (%)	65.0	78.4	0.2516
MELD score	10 [7–40]	11 [6–36]	0.3888
Etiology of liver disease			0.3226
Alcohol (%)	15.0	26.1	
Hepatitis B (%)	30.0	12.6	
Hepatitis C (%)	35.0	43.3	
NASH (%)	10.0	10.8	
Other (%)	10.0	4.5	
Within Milan criteria (%)	35.0	53.2	0.1522
AFP pre-treatment (U/mL)	101.3 [3.6–7454.0]	14.2 [1.0–13,923.0]	0.0036
Poor differentiation G3 (%)	25.0	8.1	0.0402
Microvascular invasion V1 (%)	45.0	4.5	< 0.0001
Complete necrosis (%)	5.0	52.3	< 0.0001

### Survival

**Fig. 1:**
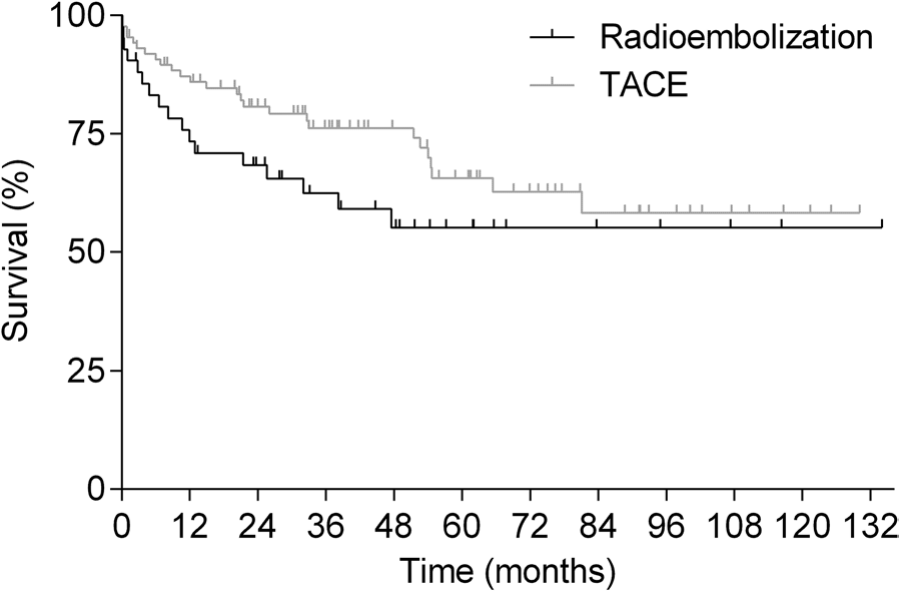
Overall survival* of patients who underwent radioembolization bridging treatment in comparison to patients who underwent TACE bridging treatment (*p* = 0.1522). *reference point for calculation of survival: day of liver transplantation (= day 0)

Median overall survival for the entire study cohort was 35.8 [0.0–134.0] months. Eighteen patients (13.7%) died due to infectious complications, 5 patients (3.9%) died due to de novo malignancy and 15 patients (11.5%) died due to recurrent HCC, while seven patients died of other causes. There was no statistical difference regarding survival between both groups of bridging treatment (Fig. 1).

### Multivariable analysis of possible risk factors for tumor recurrence and survival

In multivariable analysis, increasing age, male gender and histopathologically proven complete tumor necrosis were independently associated with decreased odds of tumor recurrence, while microvascular invasion was independently associated with increased odds of tumor recurrence. Comprehensive results of the multivariable binary logistic regression analysis are depicted in Table 3. Increasing MELD score and tumor recurrence were independently associated with increased odds of post-transplant death in multivariable analysis. Comprehensive results of the multivariable binary logistic regression analysis are depicted in Table 4.

**Table 3:** Results from multivariable logistic regression analysis of factors independently associated with recurrent hepatocellular carcinoma

	Odds ratio (OR)	95% confidence interval (CI)	*p*
Age (per 10 yrs.)	0.425	0.192, 0.941	0.035
Male gender	0.232	0.059, 0.920	0.038
AFP (log)	1.496	0.790, 2.831	0.216
Within Milan criteria	0.715	0.181, 2.819	0.632
TACE	0.480	0.116, 1.993	0.312
Poor differentiation G3	1.917	0.275, 13.370	0.511
Microvascular invasion V1	12.889	2.499, 66.483	0.002
Complete necrosis	0.067	0.007, 0.630	0.018

**Table 4:** Results from Cox regression analysis of factors independently associated with postoperative survival

	Hazard ratio (HR)	95% confidence interval (CI)	*p*
Age (per 10 yrs.)	1.635	0.990, 2.702	0.055
Male gender	1.093	0.510, 2.346	0.891
MELD score	1.062	1.020, 1.106	0.003
Donor risk index	0.576	0.253, 1.313	0.190
TACE	0.910	0.459, 1,805	0.788
Tumor recurrence	3.456	1.715, 6.964	0.001

## Discussion

In the clinical practice, given the unpredictable waiting time for liver transplantation, nearly all waitlisted HCC patients are treated with locoregional bridging treatment [28]. The aims of bridging treatment are to decrease waitlist dropout prior to transplantation and to reduce the risk of HCC recurrence post-transplant, therefore, improving overall survival. Until now, no prospective randomized studies comparing different forms of bridging treatment are available; and the true effect of bridging treatment on post-transplant tumor recurrence as well as survival remains unknown.

The effectiveness of locoregional bridging treatment on preventing post-transplant HCC recurrence and thus improving post-transplant survival appears to be limited to patients who achieve a complete pathologic response on explant pathology [29]. When treatment response, i.e., tumor necrosis, was achieved with locoregional bridging treatments, even patients with HCC exceeding the Milan criteria had demonstrated low rates of post-transplant HCC recurrence and mortality [18, 20]. On the other hand, patients with partial or no response to bridging treatment experienced a significantly higher rate of HCC recurrence after liver transplantation [30]. In our study, overall tumor recurrence was 16.5%, which is comparable to recurrence rates of 15–20% after liver transplantation in the literature [16, 31]. Among patients with complete pathologic tumor response after locoregional bridging treatment tumor recurrence was 1.8%, while it was 28.2% among patients with only partial or no response on explant specimen. Hence, it is hoped that the response to pre-transplant locoregional treatments may represent a surrogate marker of biological tumor behavior.

So far, it is not agreed upon universal criteria assessing such treatment response. As supported by Mazzaferro et al*.*, biological markers, e.g., tumor grade and microvascular invasion, may be more direct prognostic markers than nodule size and number [32]. Furthermore, up to one third of patients are misclassified as being within or beyond the Milan criteria based on imaging studies and are understaged or overstaged when pathology is reviewed [33, 34]. The impact of tumor differentiation on post-transplant outcomes has been demonstrated in previous studies and microvascular invasion has been regarded as an even stronger prognostic factor for recurrence and mortality [30, 35, 36]. A meta-analysis of results following liver resection and liver transplantation concluded that the presence of microvascular invasion in HCC is a marker of aggressive biological tumor behavior dramatically changing the disease prognosis, particularly after potentially curative therapy [36]. We were able to confirm that poor tumor differentiation as well as the presence of microvascular invasion on explant specimen were significantly more frequent among patients with HCC recurrence. In multivariable analysis, microvascular invasion was the only factor independently associated with increased odds of tumor recurrence. Hence, such patients should be followed-up carefully.

Unfortunately, microvascular invasion cannot be predicted safely on conventional imaging and is detected inconsistently on biopsy sampling. Attempts to identify surrogate biomarkers of microvascular invasion, e.g., AFP, histopathological grading, have so far failed. In our study, patients with recurrent HCC had significantly higher pre-treatment AFP levels consistent with a recent study demonstrating a better prognosis for liver transplant recipients with non AFP-producing HCC [37]. The best AFP cutoff in predicting prognosis after liver transplantation is still subject to debate. For HCC within Milan criteria, an AFP value > 1000 ng/mL was shown to be associated with worse outcome after liver transplantation [38, 39]. Patients with an AFP > 500 ng/mL at listing and especially at the time of transplant had a worse outcome irrespective of the Milan criteria [31]. Earlier studies demonstrated that patients with AFP levels ≥ 66 ng/mL prior to liver transplantation had poorer outcomes after liver transplantation independent of the Milan criteria [40]. Furthermore, AFP levels > 400 ng/mL lowered the probability of obtaining a complete response to locoregional bridging treatment [30]. Liver transplant recipients demonstrating a decrease in AFP after treatment, but not yet normalized AFP values prior to liver transplantation have shown an increased risk of tumor recurrence as well [7].

The best method of bridging treatment remains a topic of debate. In our study, patients with complete tumor necrosis on explant specimen naturally did not express microvascular invasion. As long as we may not detect the presence of microvascular invasion safely pre-transplant, one must achieve successful bridging treatment resulting in complete necrosis on explant specimen to prevent the presence of microvascular invasion. Histopathologically proven complete tumor necrosis on explant specimen was independently associated with decreased odds of tumor recurrence in our study, besides increasing recipient age and male gender.

The comparison of radioembolization and TACE when applied as bridging treatment in patients awaiting liver transplantation did not yield any significant differences in tumor recurrence and survival in our study. Both treatments seem to be equivalent for this purpose in our study cohort. We report a large number of patients beyond the Milan criteria since in Germany only pre-treatment tumor criteria are considered for organ allocation; even if downstaging is achieved clinically, these patients do not benefit from priority allocation. In the radioembolization group, only 20% of the patients met the Milan criteria due to our treatment algorithm; despite the large tumor load this treatment strategy achieved comparable rates of complete tumor necrosis as among patients with lesser tumor load treated with TACE. This finding is supported by a previous study demonstrating that radioembolization achieves downstaging from UNOS T3 to T2 in 62% of patients with a tumor size of 5–8 cm [41].

A clinical advantage of radioembolization is the significantly smaller number of pre-transplant intraarterial treatments. As a result of repeated endovascular trauma, arteritis of the celiac and hepatic arteries may complicate TACE and transplant recipients could be exposed to a significant risk of arterial complications during liver transplantation [42]. For radioembolization bridging treatment, this increased risk was not detected [43].

Compared to TACE, radioembolization resulted in a significant decrease in disease progression at 1 year as demonstrated by Lewandowski et al*.* [41]. This finding is of particular interest when waiting times for liver transplantation further increase. In a UNOS registry study, a longer waiting time for liver transplantation resulted in improved survival rates after liver transplantation for HCC patients as a longer waiting time may select liver transplant candidates with a favorable tumor biology and consequently lower risk of post-transplant mortality [44]. According to recent modifications to the Organ Procurement and Transplantation Network (OPTN) policy for granting MELD exception points to potential HCC liver transplant recipients, a mandatory 6-month waiting period before awarding 28 MELD exception points has been introduced. Yet, increased pre-transplant waiting time is significantly associated with the presence of microvascular invasion [45].

There are some limitations to our study. This is a single-center study carrying the risk of bias for treatment. In a retrospective setting, any causal relationship between type of bridging treatment and tumor recurrence as well as survival cannot be confirmed. Many confounders may occur in the analysis of post-transplant outcome of HCC patients. Nevertheless, this is the first European study comparing the effect of radioembolization and TACE on postoperative tumor recurrence and survival and may serve as an additional background for randomized controlled studies in this field.

Despite the fact that intraarterial bridging treatment is nowadays applied in most transplant centers, further studies are necessary to define the timing and the efficacy of different treatment modalities for HCC patients awaiting liver transplantation. Given what is known about the risk of waitlist drop-out among HCC patients, a randomized controlled trial comparing bridging treatment to no treatment in patients awaiting liver transplantation may be difficult to justify. A large randomized controlled trial definitely addressing the superiority of one form of locoregional bridging treatment over another may be difficult to be conducted as well due to variations in waiting times and institutional experience.

## Conclusions

Intraarterial bridging treatments may not only prevent waitlist drop-out but also can have a positive impact on HCC recurrence after liver transplantation, especially in patients with histopathologically proven complete tumor necrosis. Both radioembolization and TACE represent successful treatment strategies.

## Data Availability

All data generated or analyzed during this study are included in this published article.
